# Thermodynamic Entropy-Based Fatigue Life Assessment Method for Nickel-Based Superalloy GH4169 at Elevated Temperature Considering Cyclic Viscoplasticity

**DOI:** 10.3390/e26050391

**Published:** 2024-04-30

**Authors:** Shuiting Ding, Shuyang Xia, Zhenlei Li, Huimin Zhou, Shaochen Bao, Bolin Li, Guo Li

**Affiliations:** 1 School of Energy and Power Engineering, Beihang University, Beijing 100191, China; dst722@163.com (S.D.); xsy0902@buaa.edu.cn (S.X.); lbl123@buaa.edu.cn (B.L.); lg666@buaa.edu.cn (G.L.); 2 Research Institute of Aero-Engine, Beihang University, Beijing 100191, China; zhou_hm@buaa.edu.cn (H.Z.); advent@buaa.edu.cn (S.B.); 3 Tianmushan Laboratory, Hangzhou 310051, China

**Keywords:** low-cycle fatigue (LCF), cyclic viscoplasticity, thermodynamic entropy generation, life prediction, nickel-based superalloy

## Abstract

This paper develops a thermodynamic entropy-based life prediction model to estimate the low-cycle fatigue (LCF) life of the nickel-based superalloy GH4169 at elevated temperature (650 °C). The gauge section of the specimen was chosen as the thermodynamic system for modeling entropy generation within the framework of the Chaboche viscoplasticity constitutive theory. Furthermore, an explicitly numerical integration algorithm was compiled to calculate the cyclic stress–strain responses and thermodynamic entropy generation for establishing the framework for fatigue life assessment. A thermodynamic entropy-based life prediction model is proposed with a damage parameter based on entropy generation considering the influence of loading ratio. Fatigue lives for GH4169 at 650 °C under various loading conditions were estimated utilizing the proposed model, and the results showed good consistency with the experimental results. Finally, compared to the existing classical models, such as Manson–Coffin, Ostergren, Walker strain, and SWT, the thermodynamic entropy-based life prediction model provided significantly better life prediction results.

## 1. Introduction

Nickel-based superalloys GH4169 have similar microstructure and mechanical properties to those of Inconel 718, which is the most popular superalloy in the turbine industry [1]. The applications of GH4169 is widely used in gas turbine components due to its satisfactory elevated-temperature strength and good resistance to creep, oxidation, and corrosion [2]. The limiting temperature for GH4169 is 650 °C, within the operating temperature range (600–700 °C) for life-limited parts [3], such as disks and shafts. These life-limited parts experience heavy cyclic loading due to startup and shutdown procedures, which inevitably lead to low-cycle fatigue (LCF) damage at elevated temperatures [4]. Consequently, the LCF behavior and life prediction of nickel-based superalloys at elevated temperatures are significant concerns in ensuring the reliability and safety of turbine engines in operation.

Predicting LCF behavior and fatigue failure life (*N_f_*) of mechanical components requires a reliable constitutive description and an accurate life prediction model. For expressing the stress–strain response behavior at elevated temperature, perhaps the best-established constitutive model is the Chaboche unified viscoplastic constitutive model [5,6,7,8]. This model, originating from the Armstrong–Frederick [9] kinematic hardening rule, is well capable of modeling inelastic behaviors such as cyclic hardening/softening, the Bauschinger effect, stress relaxation, ratcheting effect, and creep. Subsequently, the Chaboche model was modified and improved to simulate emerging stress–strain behavior under more complex loading conditions. For example, Bernhart et al. [10] improved the isotropic hardening equation to describe the varying speed of cyclic softening for different loading stages at elevated temperature. Li et al. [11] modified the unified viscoplastic constitutive model to consider the influences of non-proportional hardening and dynamic strain aging on stress–strain behavior under axial–torsional thermomechanical fatigue for GH4169. Hu et al. [12] developed a modified Chaboche unified model combined with Kachanov damage evolution to describe the deformation behavior of DZ125 considering material anisotropy and anisothermal conditions. All these works suggest that the Chaboche model has satisfied robustness and flexibility to deal with various loading conditions for a wide range of materials.

Meanwhile, LCF life prediction is also critical for ensuring the operating safety of turbine components. Life prediction models for LCF regimes are mainly categorized into strain-based [13], strain energy-based [14], stress-based [15], and continuous damage mechanics (CDM) [16,17], etc. Manson and Coffin [13,18] first established their life prediction models based on strain, and then the Morrow model [19], Walker model [20], SWT model [21], etc., were proposed separately to consider different influencing factors (mean stress, load ratio, etc.). Furthermore, strain energy-based models and stress-based models also provide competitive approaches for predicting failure life. Ostergren [14] and Liu [22] innovatively defined a tensile hysteresis energy model and virtual strain energy model, respectively, to relate the fatigue failure life. Moreover, a stress-based model generally based on Basquin’s equation [23] and then various types of equivalent stress amplitude have been proposed to modify this equation [24,25] to consider ratcheting effect, mean stress, etc. CDM life prediction models stem from the non-linear fatigue damage accumulation theory proposed by Lemaitre and Chaboche [8]. Subsequently, the CDM-based model was combined with micro–macro variety [26], XFEM technology [27], and the critical plane method [28], etc., to analyze different fatigue issues. Nevertheless, in recent research, many scientists believed these approaches are largely empirical in nature and fail to unveil the physical nature of the fatigue process, such as the mechanism of damage evolution and accumulation [29]. Therefore, a series of fatigue assessment methods within the framework of thermodynamics were proposed [30] and then developed into a hot topic in fatigue fracture research.

Fatigue is a process of material degradation from nucleation and propagation of cracks that eventually causes failure. In this process, energy is irreversibly dissipated into heat due to internal friction and the system disorder continues to increase until fracture occurs. According to the second law of thermodynamics, entropy can be adopted to describe the degree of system disorder and reflect the physical mechanism of the fatigue damage process [31]. Basaran [32] first proposed a monotonic disorder qualification function based on Boltzmann probabilistic entropy for fatigue and tensile failure monitoring. Based on their work, Wang and Yao [33] proposed an entropy-based failure prediction model for creep failure. Bryant et al. [28] pointed out that entropy generation is a measure of permanent disorganization and proposed a degradation–entropy generation (DEG) methodology to characterize material system degradation. Recently, Khonsari et al. [29] proposed the concept of fatigue fracture entropy (FFE), and then Amiri [34], Naderi [35], and Liakat [36,37] confirmed that FFE is a material constant at low- and high-cycle fatigue that is independent of factors such as loading type, specimen geometry, frequency, and stress concentration. Compared with the empirical curve-fitting models, entropy-based models are more fundamental from the physical perspective of energy dissipation and hold promise to unveil underlying damage evolution and accumulation.

Recently, an entropy-based model was successfully adopted for complex conditions such as multiaxial loading [38], thermal loading [39] and wear [40]. However, considering the significance of the constitutive equation for accurately predicting the deformation behavior of nickel-based superalloys applied in a turbine engine, we believe that research on the entropy-based model requires it be combined with a suitable constitutive theory. There are few studies in the existing literature indicating that entropy-based models have been investigated for nickel-based superalloys. Therefore, the present work makes the first attempt to propose an entropy-based assessment within the framework of cyclic viscoplasticity and introduce the influence of loading ratio for GH4169 superalloys at elevated temperature (650 °C). A MATLAB program was coded to perform cyclic viscoplasticity analysis and entropy generation calculation in the chosen material system for establishing the framework of entropy-based life assessment. Then, a series of experimental data was utilized to investigate cumulative entropy generation and determine the fatigue fracture entropy (FFE) for GH4169. Finally, a novel thermodynamic entropy-based life prediction model is proposed considering the influence of load ratio, and the accuracy of this model was validated by comparing it with the verified classical life prediction models and experimental data.

## 2. Theory and Formulation

### 2.1. The Viscoplastic Constitutive Model

The unified viscoplastic constitutive model developed by Chaboche [6] was adopted to represent uniaxial cyclic behavior in this paper. Based on the small-strain hypothesis, the total strain rate ${\dot{\varepsilon }}_{t}$ can be additively divided into an elastic part ${\dot{\varepsilon }}_{e}$ and an inelastic part ${\dot{\varepsilon }}_{\mathrm{in}}$:

(1) $${\dot{\varepsilon }}_{t}={\dot{\varepsilon }}_{e\;}+{\dot{\varepsilon }}_{\mathrm{in}}$$

The elastic strain and total stress are subjected to Hooke’s law:

(2) $$\dot{\sigma }=E{\dot{\varepsilon }}_{e\;}=E({\dot{\varepsilon }}_{t}-{\dot{\varepsilon }}_{\mathrm{in}})$$

where *E* is Young’s modulus and *σ* is the stress. A viscoplastic potential (Ω) is defined as:

(3) $$\Omega =\frac{Z}{n^{\prime }+1}{\left\langle \;\frac{f}{Z}\;\right\rangle }^{n\prime +1}$$

where the symbol 〈 〉 is Heaviside function, defined as $\left\langle x\right\rangle =x\;\mathrm{if}\;x\;>\;0$ and $\left\langle x\right\rangle =0\;\mathrm{if}\;x\;\leq \;0$. *Z* and *n*′ stand for viscous parameters. The yield function *f* is defined as:

(4) $$f=J(\sigma -x)-R-k_{0}$$

where *R*, *X*, and *k*_0_ are the drag stress, the back stress and the initial yield stress, respectively. *J* denotes the scalar equivalent of deviatoric stress state:

(5) J(σ−X)=|σ−X|

Plastic flow occurs when *f* = 0 or $\frac{\partial f}{\partial \sigma }:\dot{\sigma }\;>\;0$. For the viscoplastic constitutive model, the stress in excess of the yield surface is acceptable and often termed “overstress”. The inelastic strain rate ${\dot{\varepsilon }}_{\mathrm{in}}$ can be determined from the flow rule as:

(6) $${\dot{\varepsilon }}_{\mathrm{in}\;}=\frac{\partial \Omega }{\partial \sigma }=\dot{p}\frac{\partial f}{\partial \sigma }={\left\langle \frac{f}{Z}\right\rangle }^{n\prime }\mathrm{sgn}\left(\sigma -X\right)$$

and:

(7) $$\mathrm{sgn}(x)=\left\{\begin{array}{cc} 1, & x>0\\ 0, & x=0\\ -1, & x<0 \end{array}\right.$$

where $\dot{p}$ is the accumulated inelastic strain rate, given by:

(8) $$\dot{p}={\left\langle \;\frac{f}{Z}\;\right\rangle }^{n\prime }=\left|{\dot{\varepsilon }}_{\mathrm{in}}\right|$$

Non-linear kinematic hardening is related to back stress, which can be obtained by accumulating each component $\left(X=\sum_{i}^{m}X_{i}\right)$ with the same evolution law. To describe the cyclic stress–strain hysteresis, two components are sufficient, and the back stress can be expressed as:

*X* = *X*_1_ + *X*_2_ (9)

and the evolution of each component can be expressed as:

(10) $$\left\{\begin{array}{c} {\dot{X}}_{1}\;=\;C_{1}a_{1}{\dot{\varepsilon }}_{\mathrm{in}}\;-\;C_{1}X_{1}\dot{p}\\ {\dot{X}}_{2}\;=\;C_{2}a_{2}{\dot{\varepsilon }}_{\mathrm{in}}\;-\;{C_{2}X}_{2}\dot{p} \end{array}\right.$$

where the asymptotic value of each component is given by *a*_1_ and *a*_2_. *C*_1_ and *C*_2_ indicate the speed to reach *a*_1_ and *a*_2_, respectively. The unified equation in Equation (10) can be expressed as ${\dot{X}}_{m}=C_{m}a_{m}{\dot{\varepsilon }}_{\mathrm{in}}\;-\;{C_{m}X}_{m}\dot{p}$ with a linear kinematic hardening term ($C_{m}a_{m}{\dot{\varepsilon }}_{\mathrm{in}}$) and a dynamic recovery term (${C_{m}X}_{m}\dot{p}$), respectively. Generally, *X*_1_ can be used to determine the initial growth stage of stress after reaching the initial yield stress *k*_0_, and *X*_2_ is used to find the asymptotic of the cyclic stress–strain hysteresis. Then, the isotropic hardening rule controls the evolution of drag stress *R* as:

(11) $$\dot{R}=b(Q\;-\;R)\dot{p}$$

where *b* is the speed towards the saturation and *Q* is the asymptotic value of *R*.

The rate dependency of the stress is described by Norton’s creep law [41,42] as:

(12) $$\sigma_{v}\;=\;Z{\dot{p}}^{1/n\prime }$$

where $\sigma_{v}$ is the viscous overstress, and the applied stress σ can be decomposed as:

(13) $$\sigma =X\;+\;(R\;+\;k_{0}+\sigma_{v})\mathrm{sgn}(\sigma -X)$$

**Figure 1:**
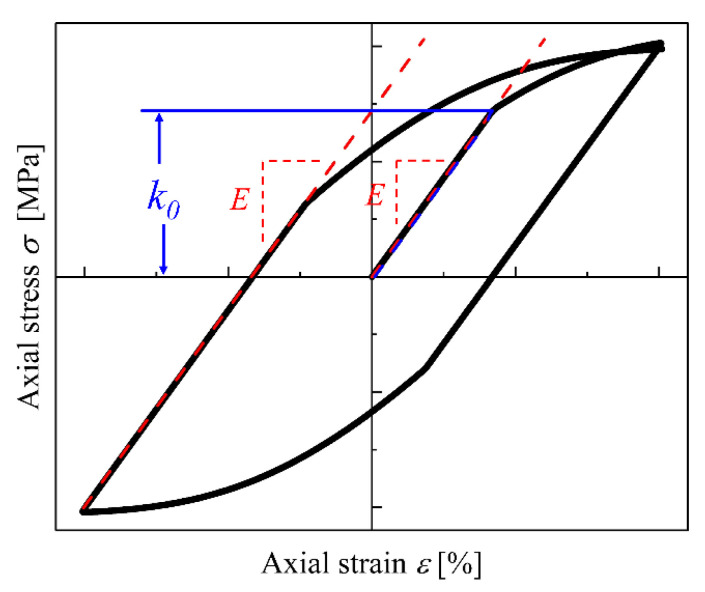
Diagram to obtain Young’s modulus *E* and the value of *k*_0_ in the first cyclic curve.

The model in the present work contains 10 material parameters, namely, *E*, *k*_0_, *b*, *Q*, *C*_1_, *a*_1_, *C*_2_, *a*_2_, *Z* and *n*′. Subsequently, a brief description of how to obtain these parameters is given below. Young’s modulus *E* is the slope the of the initial linear region in the first cyclic curve, and *k*_0_ stands for the stress value that firstly deviates from the initial linear region and is approximately equal to the stress that causes 0.01% inelastic strain. Figure 1 presents the identification of parameters *E* and *k*_0_. Then, the parameters *b* and *Q* can be determined by integration of Equation (11) as:

(14) $$R=Q(1-e^{-\mathrm{bp}})$$

where the value of *b* is the gradient in the fit curve of ln(1 − *R*/*Q*) vs. *p* (≈2*N*∆*ε_in_*) and *Q* is the difference in stresses between the first cycle and the saturation.

The estimation of parameters *C*_1_, *a*_1_, *C*_2_ and *a*_2_ can be obtained from the tensile part of the first cyclic curve. Equation (10) can be integrated to give the following expressions:

(15) $$X_{m}=a_{m}\left(1-e^{-C_{m}\varepsilon_{\mathrm{in}}}\right),\;m=1,\;2$$

Substituting Equation (15) into Equation (13) and differentiating Equation (13) with respect to ${\dot{\varepsilon }}_{\mathrm{in}}$, the logarithm form follows:

(16) $$\ln(\frac{\partial \sigma }{\partial \varepsilon_{\mathrm{in}}}-\frac{\partial R}{\partial \varepsilon_{\mathrm{in}}})\;=\ln\left(a_{2}C_{2}\right)-C_{2}\varepsilon_{\mathrm{in}}$$

where the effects of *X*_1_ with *a*_1_ and *C*_1_ are neglected in the larger inelastic strain region. The fitting line of ln(∂*σ*/∂*ε_in_* − ∂*R*/∂*ε_in_*) vs. *ε_in_* is utilized to identify *a*_2_ from the y-axis intercept and *C*_2_ from the slope of the line. Similarly, parameters *a*_1_ and *C*_1_ can be determined by the intercept and the slope from data in the ln(∂*σ*/∂*ε_in_* − ∂*R*/∂*ε_in_* − ∂*X*_2_/∂*ε_in_*) vs. *ε_in_* plot:

(17) $$\ln(\frac{\partial \sigma }{\partial \varepsilon_{\mathrm{in}}}-\frac{\partial R}{\partial \varepsilon_{\mathrm{in}}}-\frac{\partial X_{2}}{\partial \varepsilon_{\mathrm{in}}})=\ln(a_{1}C_{1})-C_{1}\varepsilon_{\mathrm{in}}$$

Finally, the parameters *Z* and *n*′ can be determined from the data of multiple hardening, relaxation tests, and creep tests. The overstress can be expressed as $\ln(\sigma_{v})\;=\ln({\dot{\varepsilon }}_{\mathrm{in}})/n^{\prime }+\ln Z$, and the value of *Z* and *n*′ can be obtained from the intercept and slope in the fitted line of $\ln(\sigma_{v})$ vs. $\ln({\dot{\varepsilon }}_{\mathrm{in}})$. More detailed descriptions can be found in Tong’s representative work [42]. The constitutive parameters of GH4169 at 650 °C adopted in this paper are collected from Nanjing University of Aeronautics and Astronautics’ constitutive experiments, as shown in Table 1.

### 2.2. Thermodynamic Analysis of Fatigue

**Figure 2:**
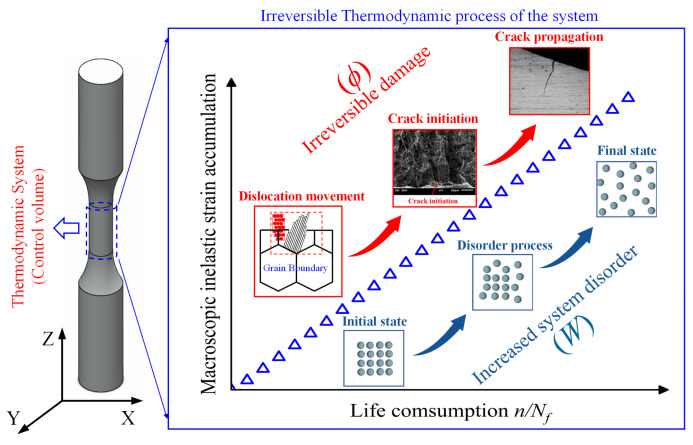
Irreversible thermodynamic failure mechanism of a material system during the fatigue process.

The irreversible process of the thermodynamic system (control volume) in fatigue failures, including irreversible inelastic strain accumulation, dislocation movement, crack initiation and propagation, etc., is shown in Figure 2. These irreversible phenomena increase the disorder of the system, which is inevitably accompanied by energy dissipation and entropy generation accumulation. To demonstrate the relationship between entropy and disorder, Boltzmann [44] first used statistical mechanics to give a precise meaning to the disorder and established a connection between the disorder and entropy as:

(18) S=klnW

where *k* is the Boltzmann constant in J/K and *W* is the dimensionless disorder parameter. Then, the disorder parameter with entropy per unit mass $s_{m}$ (J/kg∙K) can be expressed as:

(19) $$W=e^{(m_{s}/N_{a}k)s_{m}}$$

(20) $$k=\frac{R_{g}}{N_{a}}$$

where *m_s_*, *N_a_* and *R_g_* are the molar mass of the current material (kg/mol), Avogadro’s number (mol^−1^), and the gas constant (J/mol∙K), respectively. According to Basaran’s work [32], the increase in disorder can be defined as an increasing damage parameter ϕ, expressed as:

(21) $$\phi \;=\phi_{\mathrm{cr}}\frac{W-W_{0}}{W_{0}}=\phi_{\mathrm{cr}}\left(1-e^{-(m_{s}/R_{g})\left(s_{m}-s_{m}^{0}\right)}\right)$$

where *W*_0_ is the disorder corresponding to the initial state with initial entropy $s_{m}^{0}\left(≅\;0\right)$ and $\phi_{\mathrm{cr}}$ is a proportional critical disorder coefficient that can be determined from experiments. Equation (21) can be a basic equation to model fatigue, tensile, and even creep failure [32,33], and the core in Equation (21) is the value of $s_{m}-s_{m}^{0}$ during the failure process as:

(22) $$s_{m}-s_{m}^{0}=\int_{t_{0}}^{t}\frac{d\delta }{\rho \mathrm{dt}}\mathrm{dt}$$

where ρ is the density in kg/m^3^. Density can be used to calculate $s_{m}$ from the entropy generation in unit volume (J/m^3^∙K). It can be seen that the entropy in statistical physics and in the thermodynamic sense are equivalent, and the key is to calculate the cumulative entropy generation. Thus, this paper directly uses increasing entropy generation to represent increasing damage in the fatigue failure process, as suggested by Khonsari et al. [29,34,35,36,37].

Combining with classical thermodynamics, the first and second laws of thermodynamics were formulated to describe irreversible processes and then applied to the control volume system in specimens. According to the first law of thermodynamics, the total energy change ΔU within an arbitrary control volume can change only if energy flows into (or out of) the control volume through its boundary, and is expressed as:

(23) ΔU=δQ + δW

where δ*Q* and Δ*W* are the change in supplied heat and the mechanical work done on the control volume, respectively. The unit in Equation (23) is J and the specific quantity (per volume) of Equation (23) is as follows:

(24) $$\rho \frac{\mathrm{du}}{\mathrm{dt}}=\left(\dot{q}-\mathrm{div}J_{q}\right)+\sigma :{\dot{\varepsilon }}_{t}$$

where *u* (J/m^3^) is the internal energy per unit volume, $\dot{q}$ (J/m^3^∙s) is the rate of heat generation by a heat source, *Jq* (J/m^2^∙s) is the rate of heat flux across the boundary, *Jq* = −λgradT, and λ is the heat conduction coefficient in W/m·K. The second term on the right-hand side (RHS) of Equation (24) is the rate of mechanical work per unit volume. According to Clasius-Duhem inequality, the change of entropy is:

(25) $$\mathrm{ds}=\frac{\delta Q_{\mathrm{irr}}}{T}+d\delta$$

where ${\delta Q}_{\mathrm{irr}}$ is the change in irreversible heat dissipation per volume (J/m^3^) and δ denotes the entropy generation per volume in J/m^3^∙K. According to Clausius–Duhem inequality, Equation (25) can be written as:

(26) $$\frac{d\delta }{\mathrm{dt}}=\frac{\mathrm{ds}}{\mathrm{dt}}\;-\;\left[\frac{\dot{q}}{T}\;-\;\mathrm{div}(\frac{J_{q}}{T})\right]\geq 0$$

where:

(27) $$\mathrm{div}(\frac{J_{q}}{T})=\frac{\mathrm{div}J_{q}}{T}-\frac{J_{q}\cdot \mathrm{gradT}}{T^{2}}$$

Then, Helmholtz free energy Ψ is defined as Ψ = *u − Ts* and then rewritten by taking the time derivative of both sides:

(28) $$\frac{\mathrm{du}}{\mathrm{dt}}=\frac{\partial \Psi }{\partial t}+s\frac{\mathrm{dT}}{\mathrm{dt}}+T\frac{\mathrm{ds}}{\mathrm{dt}}$$

Ψ is also the state function dependent on the elastic strain $\varepsilon_{e}$, temperature *T* and internal material variables *V_k_*:

(29) $$\Psi \;=\Psi (\varepsilon_{e},T,V_{k})$$

Using the chain rule, the rate of Helmholtz free energy is obtained:

(30) $$\frac{d\Psi }{\mathrm{dt}}=\frac{\partial \Psi }{\partial \varepsilon_{e}}:{\dot{\varepsilon }}_{e}+\frac{\partial \Psi }{\partial T}\dot{T}+\frac{\partial \Psi }{\partial V_{k}}{\dot{V}}_{k}$$

where $\frac{\partial \Psi }{\partial V_{k}}$ is the force of internal variables. Substituting Equation (30) into Equation (28) yields:

(31) $$\frac{\mathrm{du}}{\mathrm{dt}}=[\frac{\partial \Psi }{\partial \varepsilon_{e}}:{\dot{\varepsilon }}_{e}+\frac{\partial \Psi }{\partial T}\dot{T}+\frac{\partial \Psi }{\partial V_{k}}{\dot{V}}_{k}]+s\frac{\mathrm{dT}}{\mathrm{dt}}+T\frac{\mathrm{ds}}{\mathrm{dt}}$$

In the framework of small strains, we have $\sigma =\rho \frac{\partial \Psi }{\partial \varepsilon_{e}}$, $s=-\frac{\partial \Psi }{\partial T}$, and $A_{k}=\rho \frac{\partial \Psi }{\partial V_{k}}{\dot{V}}_{k}$. Thus, Equation (31) can be expressed as:

(32) $$\frac{\mathrm{du}}{\mathrm{dt}}=\sigma :\;{\dot{\varepsilon }}_{e}+A_{k}{\dot{V}}_{k}+T\frac{\mathrm{ds}}{\mathrm{dt}}$$

Thus, by substituting Equation (24) into Equation (32) as:

(33) $$\left(\dot{q}-\mathrm{div}J_{q}\right)\;+\sigma :\;{\dot{\varepsilon }}_{t}=\sigma :\;{\dot{\varepsilon }}_{e}+A_{k}{\dot{V}}_{k\;}+T\frac{\mathrm{ds}}{\mathrm{dt}}$$

Substituting Equations (33) and (27) into Equation (33), the entropy generation can be expressed as:

(34) $$\frac{d\delta }{\mathrm{dt}}=\frac{\mathrm{ds}}{\mathrm{dt}}-[\frac{\dot{q}}{t}-\mathrm{div}(\frac{J_{q}}{T})]=\frac{\mathrm{ds}}{\mathrm{dt}}-[\frac{\dot{q}}{T}-\frac{\mathrm{div}J_{q}}{T}+\frac{J_{q}\mathrm{gradT}}{T^{2}}]$$

Substituting Equation (34) into Equation (33), we can arrive at:

(35) $$\frac{d\delta }{\mathrm{dt}}=\frac{\sigma :\;{\dot{\varepsilon }}_{\mathrm{in}}}{T}-\frac{A_{k}{\dot{V}}_{k}}{T}-\frac{J_{q}\mathrm{gradT}}{T^{2}}$$

This is the general expression of the entropy generation rate in the control volume of the specimen. On the RHS of Equation (35), the first term is entropy generation due to irreversible inelastic deformation, the second term stands for entropy generation related to internal variables, and the third term represents entropy generation due to heat conduction. In this paper, a specimen’s temperature is considered equal in isothermal LCF tests, and the axial temperature gradient (along the Z-direction of the specimen, as shown in Figure 2) $\frac{∆T}{∆Z}$ is the main temperature gradient direction for heat conduction, which can be assumed to be 0 (∆T = 0). Thus, the third term on the RHS of Equation (35) can be neglected and is approximately equal to 0. The proof of this phenomenon in isothermal tests also can be found in [45,46]. Thus, for isothermal tests, irreversible entropy generation for specimens under an external applied load is assumed to be mainly dependent on the dissipation caused by irreversible inelastic deformation ($\sigma :{\dot{\varepsilon }}_{\mathrm{in}}$) and internal variables ($A_{k}{\dot{V}}_{k}$). Thus, the composition of irreversible inelastic deformation and the specific form of the internal variables is discussed in the following content.

## 3. Entropy Generation Modeling in Viscoplastic Framework

### 3.1. Entropy Generation Rate Model

Based on the above thermodynamic analysis, a unified viscoplastic constitutive model is introduced to specify Equation (35) considering irreversible deformation mechanisms for an extension of the theory. Theoretically, the inelastic strain rate in the framework of viscoplasticity can be separated as plastic part ${\dot{\varepsilon }}_{p}$ and viscous (creep) part ${\dot{\varepsilon }}_{v}$. Therefore, the inelastic strain rate ${\dot{\varepsilon }}_{\mathrm{in}}$ can be rewritten as:

(36) $${\dot{\varepsilon }}_{\mathrm{in}}={\dot{\varepsilon }}_{t}-{\dot{\varepsilon }}_{e\;}={\dot{\varepsilon }}_{p}+{\dot{\varepsilon }}_{v}$$

It should be noted that the proportion of viscous (creep) part is small under the loading conditions in this study. A viscous (creep) effect may become significant when the loading cycle has a longer cycle time or dwell time, and the damage failure mechanism changes from a cycle-dependent fatigue damage mechanism to a time-dependent creep damage mechanism, as proved in [47].

Subsequently, internal variables require specific definitions to characterize the irreversible deformation mechanisms related to cyclic hardening/softening or microstructural variation. According to Haghshenas’s description [48], hardening/softening can characterize the energy stored in a material due to dislocation. In the first few cycles, dislocations multiply and pile up to overcome barriers, causing inelastic deformation with isotropic hardening. Then, dislocation multiplication and annihilation reach equilibrium when the hardening term is mainly dominated by kinematic hardening. Therefore, Equation (29) can be modified by decoupling elastic behavior and hardening as follows:

(37) $$\Psi \;=\Psi (\varepsilon_{e},T)\;+\Psi (r,\alpha ,m_{k},T)$$

where *r*, *α* and *m_k_* represent the isotropic hardening variable (*V*_1_), kinematic hardening variable (*V*_2_), and other internal variables (*V_m_*). Their conjugates’ thermodynamic forces are *A*_1_ *= R = ρ*(*∂*Ψ*/∂r*), *A*_2_ *= X = ρ*(*∂*Ψ*/∂α*) and *A_k_ = M_k_ = ρ*(*∂*Ψ*/∂m_k_*), respectively. *R*, *X* and *M_k_* are the drag stress, the back stress, and other corresponding thermodynamic forces, respectively. The other thermodynamic forces can be internal friction, dislocation motions, phase transformation, etc., and are mainly adapted to the life assessment for specimens loaded under yield stress, which can cause high-cycle fatigue (HCF). In the LCF regime, *R* and *X* can be the main thermodynamic forces as *A*_1_ and *A*_2_. Then, according to the evolution functions in Equations (10) and (11), $\varepsilon_{\mathrm{in}}$ and *p* are the corresponding strain parameters for defining thermodynamic flows (*r* and *α*). Therefore, the specific forms of *r* and *α* related to $\varepsilon_{\mathrm{in}}$ and *p* can be expressed as [8]:

(38) $${\dot{V}}_{1}=\dot{r\;}=\dot{p}=\left|{\dot{\varepsilon }}_{\mathrm{in}}\right|$$

(39) $${\dot{V}}_{2\;}={\dot{\alpha }}_{m}={\dot{\varepsilon }}_{\mathrm{in}}-\left(\frac{X_{m}}{a_{m}}\right)\dot{p},\;m=2$$

the corresponding entropy generation rate for isotropic hardening can be written in the following forms:

(40) $$\frac{A_{1}{\dot{V}}_{1}}{T}=\frac{R\dot{r}}{T}$$

and the entropy generation rate for kinematic hardening, expressed as:

(41) $$\frac{A_{2}{\dot{V}}_{2}}{T}=\frac{X\dot{\alpha }}{T}=\frac{X_{1}{\dot{\alpha }}_{1}}{T}+\frac{X_{2}{\dot{\alpha }}_{2}}{T}$$

Meanwhile, the entropy generation due to heat conduction is assumed to be neglected in this paper, as discussed in Section 2.2. The entropy generation rate in Equation (33) can be rewritten as:

(42) $${\dot{\delta }}_{g}=\frac{\sigma :{\dot{\varepsilon }}_{\mathrm{in}}}{T}-\frac{R\dot{r}}{T}-\frac{X\dot{\alpha }}{T}=\frac{(\left|\sigma -X\right|-R\;-\;k_{0})\left|{\dot{\varepsilon }}_{\mathrm{in}}\right|}{T}+\frac{k_{0}\left|{\dot{\varepsilon }}_{\mathrm{in}}\right|}{T}+\frac{X^{2}\left|{\dot{\varepsilon }}_{\mathrm{in}}\right|/\alpha }{T}$$

This is the entropy generation rate model in the viscoplastic framework.

**Figure 3:**
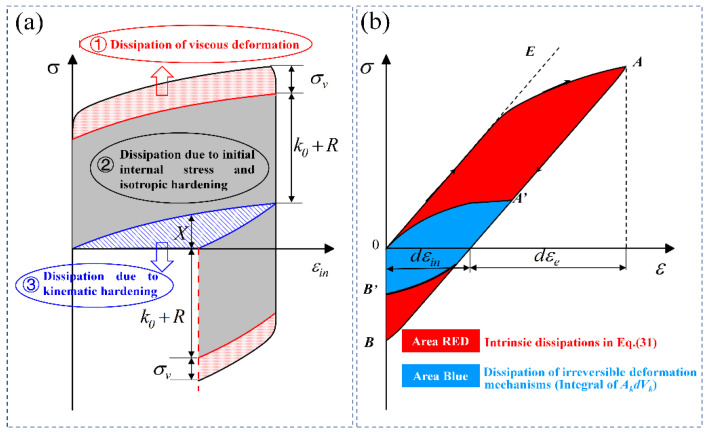
Diagram for indicting intrinsic dissipation. (**a**) Different dissipation components in the stress-inelastic strain curve; (**b**) hysteresis loop for indicating intrinsic dissipation.

In addition, parts in Equation (42) can be clarified from the perspective of energy dissipation. The numerators in the far RHS of Equation (42) are the intrinsic dissipations, each component of which is shown in the stress-inelastic curve in Figure 3a. In addition, the intrinsic dissipations (red area) can also be calculated in the hysteresis loop, as shown in Figure 3b. Therefore, the method for calculating entropy generation can be based on stress–strain responses and hardening variables.

### 3.2. Entropy Generation Accumulation

The concept of energy dissipation makes the entropy-based model similar to strain energy models. However, most of the strain energy models are essentially empirical due to their fitting relationship between experiment lives and strain energy density at the half-life. The entropy-based models focus on the accumulative process, and entropy generation is the only indicator in the thermodynamic framework to relate the irreversibility of fatigue damage evolution [49]. Naturally, the change in entropy generation during the fatigue process is a monotonic accumulation process, and the hypothesis of fatigue failure life being related to the entropy generation limit is utilized in this paper [33]. Therefore, the calculation of entropy generation accumulation in fatigue is important. To reflect the cycle characteristic of the fatigue process, a cyclic entropy generation rate (entropy generation accumulation per cycle) is defined as:

(43) $${\dot{S}}_{g}^{\;N}=\int_{0}^{t_{N}}{\dot{\delta }}_{g}\mathrm{dt}$$

where *t_N_* is the cycle time. Then, the integral of Equation (43) with respect to cycles provides the accumulation of entropy generation, expressed as:

(44) $$s_{g}^{\;N}=\int_{0}^{N_{\mathrm{tot}}}{\dot{S}}_{g}^{N}\mathrm{dN}$$

where *N_tot_* is the total number of cycles. When *N_tot_* reaches fatigue failure life *N_f_*, entropy generation accumulation can be defined as a parameter call—FFE $\left(S_{g}^{N_{f}}\right)$.

Researchers [26,27] have suggested that $S_{g}^{N_{f}}$ is a material constant that can provide a threshold to monitor fatigue damage of material. However, the influence of load ratio (*R_load_*) on entropy generation under cyclic loading was neglected in these previous works. Extensive literature has demonstrated that the load ratio can significantly affect the fatigue failure life of nickel-based superalloys at elevated temperatures [50,51,52]. This present work found that the load ratio can also affect the value of entropy generation before reaching a stable state. Under the same applied load, the initial damage becomes more severe as the load ratio increases. This paper believed that entropy generation accumulation is available to simultaneously evaluate the fatigue failure life and introduced the influence of load ratio. Therefore, the framework of an entropy-based life assessment and the influence of load ratio are discussed in the following two sections.

## 4. Proposed Thermodynamic Entropy-Based Life Assessment Framework

### 4.1. Cyclic Viscoplasticity Numerical Algorithm

**Figure 4:**
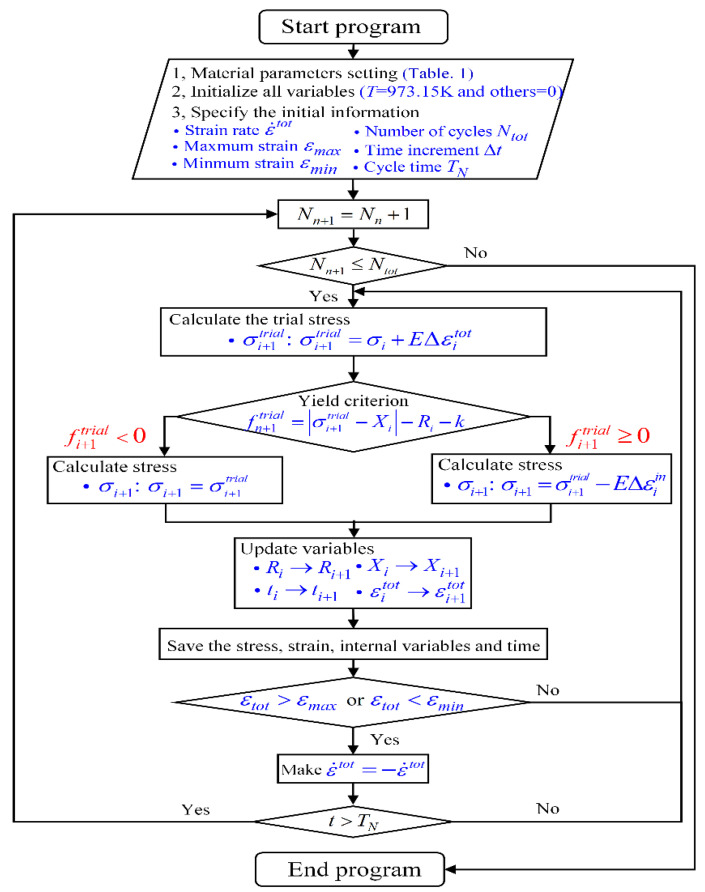
Flowchart of the MATLAB program for calculating cyclic stress–strain response.

The primary concern in this work is how to accurately obtain the cyclic stress–strain response. Generally, the specimen for simulations can be simplified as a single cubic element subjected to a near-uniform macroscopic stress–strain state. For simplicity, MATLAB language was chosen for this work to write an explicit incremental algorithm for simulating the specimen under uniaxial cyclic loading. This method eliminates the process of modeling and secondary development and improves the computational efficiency compared to simulation with commercial finite element software (Abaqus, Ansys, etc.). For instance, a calculation of 1000 cycles took only about 5 s with appropriate step-increment settings without the process of modeling and secondary development. The flowchart of this MATLAB program with version of 2020b is shown in Figure 4.

With constant strain rate ${\dot{\varepsilon }}^{t}$ and given time increment $\Delta t_{i}=t_{i+1}-\;t_{i}$, the strain increment is $\Delta \varepsilon_{i}^{t}=\Delta t_{i}{\dot{\varepsilon }}_{i}^{\;t}$. The inelastic strain increment is calculated as the following:

(45) $$\Delta \varepsilon_{i}^{\mathrm{in}}={\dot{\varepsilon }}_{i}^{\mathrm{in}}\Delta t_{i}$$

where ${\dot{\varepsilon }}_{i}^{\mathrm{in}}$ is the inelastic strain rate at *t_i_* and ${\dot{\varepsilon }}_{i}^{\mathrm{in}}$ can be expressed as:

(46) $${\dot{\varepsilon }}_{i}^{\mathrm{in}}={\left\langle \;\frac{f_{i+1}}{K}\;\right\rangle }^{n}\mathrm{sgn}\left(\sigma_{i}-X_{i}\right)$$

where *f_i+_*_1_, *σ_i_*, *X_i_* and *R_i_* are the yield function, stress, back stress and drag stress at *t_i_*, respectively. To obtain stable calculation results, the value of $\Delta t_{i}$ was set to 0.05, which means a step increment of total strain $\Delta \varepsilon_{i}^{t}$ in the order of 10^−4^. Then, a return mapping method was implemented in this algorithm that included elastic prediction and inelastic correction. Firstly, if strain increment during $\Delta t_{i}$ will not form a new inelastic increment ($\Delta \varepsilon_{i}^{\mathrm{in}}=0$), trial stress $\sigma_{i+1}^{\mathrm{trial}}$ was proposed for the elastic prediction as:

(47) $$\sigma_{i+1}^{\;\mathrm{trial}}=E(\varepsilon_{i+1}^{t}-\varepsilon_{t}^{\mathrm{in}})\;=\sigma_{i\;}+E\Delta \varepsilon_{i}^{t}$$

A trial yield criterion for the step [*t_i_*, *t_i+_*_1_] can be written as:

(48) $$f_{i+1}^{\mathrm{trial}}=\left|\sigma_{i+1}^{\mathrm{trial}}-X_{i}\right|-R_{i}-k_{0}$$

According to Equation (48), if $f_{i+1}^{\mathrm{trial}}\;<\;0$, the stress can be updated as:

(49) $$\sigma_{i+1}=\sigma_{i+1}^{\mathrm{trial}}$$

Also, if $f_{i+1}^{\mathrm{trial}}\;\geq \;0$, the trial stress requires an inelastic correction to update stress to consider increased inelastic strain through:

(50) $$\sigma_{i+1}=\sigma_{i+1}^{\mathrm{trial}}-E\Delta \varepsilon_{i}^{\mathrm{in}}$$

The value of $f_{i+1}$ in Equation (48) is approximately equal to $f_{i+1}^{\mathrm{trial}}$ in uniaxial form, and then the variables for isotropic and kinematic hardening can be updated by:

(51) $$R_{i+1}=R_{i}+\Delta R_{i}$$

(52) $$X_{i+1}=X_{i}+\sum_{m=1}^{2}\Delta X_{i}^{m}$$

Increments of drag stress $\Delta R_{i}$ and back stress $\Delta X_{i}^{m}$ are given through:

(53) $$\Delta R_{i}=b(Q-R_{i})|\Delta \varepsilon_{i}^{\mathrm{in}}|$$

(54) $$\Delta X_{i}^{\;m}=C_{m}a_{m}\Delta \varepsilon_{i}^{\mathrm{in}}-C_{m}X_{i}^{\;m}|\Delta \varepsilon_{i}^{\mathrm{in}}|$$

**Figure 5:**
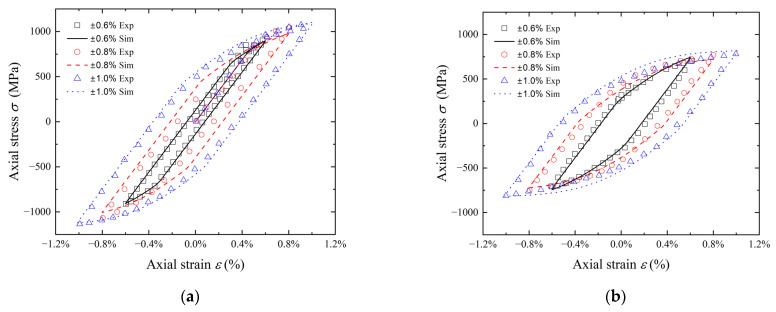
Comparison of the stress–strain hysteresis loops from simulations and experiments undergoing cyclic strain loading at 650 °C. (**a**) The first loading cycle. (**b**) The stable cyclic hysteresis loops [43].

To validate the reliability of this program, the test data of GH4169 at 650 °C [43] were utilized for comparison with the simulation results. All the test data were collected from strain-controlled uniaxial tests with a specific strain rate ($d\varepsilon /dt=0.0001\;s^{-1}$) and load ratio (*R_load_ =* −1), and the comparisons are shown in Figure 5. The figures show the initial (Figure 5a) and half-life cycles (Figure 5b) with a strain range of ±0.60%, ±0.80% and ±1.00%, respectively. Obviously, there is a small discrepancy between simulated and experimental loops. Therefore, it can be proved that this program can provide a reliable prediction of the cyclic stress–strain response.

### 4.2. Entropy Generation Calculation and Life Assessment Method

After calculating the cyclic stress–strain response, entropy generation accumulation is investigated in this section. According to the entropy generation rate model in Section 3.1, the entropy generation accumulation within $\Delta t_{i}$ can be calculated utilizing the variables stored for each step in the MATLAB program, which follows:

(55) $$\Delta \delta_{i}^{g}=\left[\left(\left|\sigma_{i+1}-\sum_{m=1}^{2}X_{i+1}^{m}\right|-R_{i+1}+\sum_{m=1}^{2}\frac{X_{i+1}^{m}}{a_{m}}\right)\left|\Delta \varepsilon_{i}^{\mathrm{in}}\right|\right]/T$$

**Figure 6:**
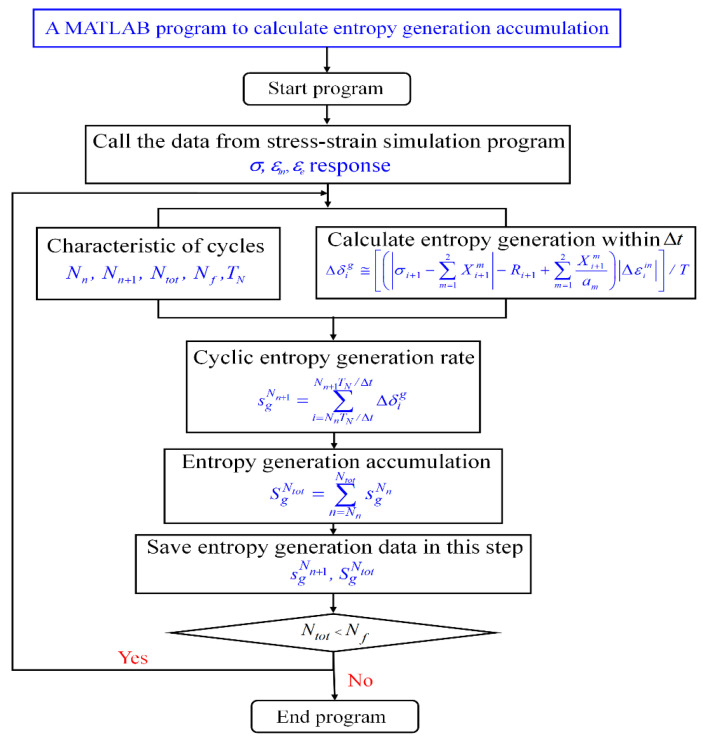
Diagram for the calculation of entropy generation accumulation in fatigue process.

**Figure 7:**
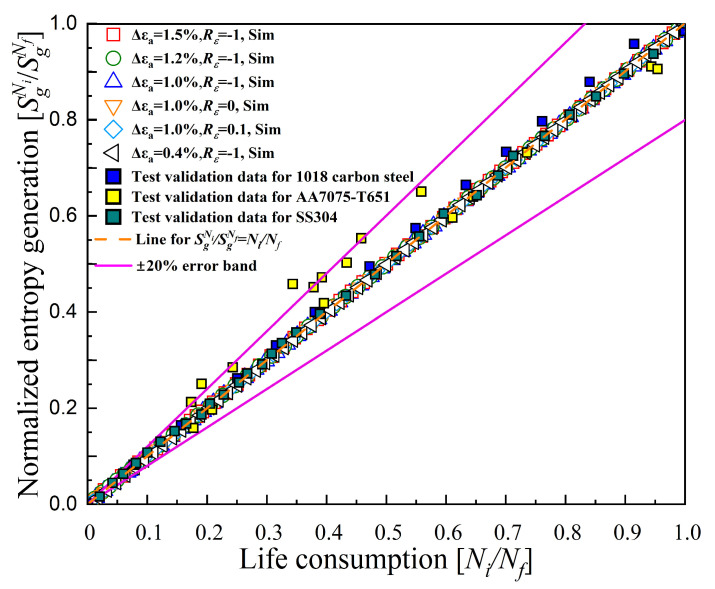
The linear relationship for normalized entropy generation ($S_{g}^{N_{i}}/S_{g}^{N_{f}}$) and life consumption ($N_{i}/N_{f}$) in the entire fatigue process with test validations.

In each cycle, the cyclic entropy generation rate (the accumulation of entropy generation within a cycle) can be calculated by:

(56) $$s_{g}^{N_{i}}=\sum_{i=N_{i-1}T_{N}/\Delta t}^{N_{i}T_{N}/\Delta t}\Delta \delta_{i}^{g}$$

where *i* is the index for the number of cycle *N* starts from *N*_0_ (*N*_0_ = 0). The entropy generation accumulation $s_{g}^{\;N_{\mathrm{tot}}}$ from *N*_0_ until a specific total cycle *N_tot_* can be calculated by:

(57) $$s_{g}^{N_{\mathrm{tot}}}=\sum_{i=N_{0}}^{N_{\mathrm{tot}}}s_{g}^{N_{i}}$$

where *N_tot_* = *N_f_*. Equation (57) represents the FFE $s_{g}^{\;N_{f}}$. The procedure for this calculation method of entropy generation accumulation in MATLAB is illustrated in Figure 6. The ideal relationship of normalized entropy generation ($s_{g}^{\;N_{i}}/s_{g}^{\;N_{f}}$) versus life consumption to failure ($N_{i}/N_{f}$) from simulations is shown in Figure 7. In real LCF tests, the saturated stage occupied most of the fatigue process (85–90%). However, the states of stress and strain may show obvious variations at the final stage, i.e., in the last 5–10% of the total life, and the strain energy density relating to the energy dissipation will gradually decrease in the final stage [53]. Despite there being a non-linear relationship at the beginning and the final stages, it can be assumed that the ideal linear relationship (orange dashed line in Figure 7) can approximately represent the whole fatigue process. Consequently, ($s_{g}^{\;N_{i}}/s_{g}^{\;N_{f}}$) versus ($N_{i}/N_{f}$) in the whole fatigue process can be expressed as:

(58) $$\frac{S_{g}^{N_{i}}}{S_{g}^{N_{f}}}=\frac{N_{i}}{N_{f}}$$

Then, three groups of isothermal fatigue test data were used with investigations of entropy generation to verify the linear relationship. The test data were from 1018 carbon steel under uniaxial tension-compression fatigue tests [54], Al7075-T651 under bending fatigue tests [55], and SS304 under torsion tests [30]. It can be seen that all the scatter points for the test data fall into ±20% error band, and the results representing this linear relationship are independent of material and loading mode. Thus, Equation (58) can represent the relationship between entropy generation and number of cycles in the entire fatigue test process. Thus, the fatigue failure life *N_f_* can be estimated as:

(59) $$N_{f}=\left[\frac{N_{i}}{S_{g}^{\;N_{i}}}\right]S_{g}^{\;N_{f}}$$

This is the linear thermodynamic relationship for the further establishment of a life prediction model, and the two key issues are the values of FFE ($S_{g}^{\;N_{f}}$) and ($N_{i}/S_{g}^{\;N_{i}}$).

## 5. Results and Discussion

This section is organized into three main subsections. The first describes the determination of FFE for GH4169 at 650 °C. Then, the effect of loading ratio *R_load_* on entropy generation accumulation is investigated. Finally, a thermodynamic entropy-based life prediction model is proposed to estimate fatigue life, and its performance is compared to both experimental fatigue life and other classical life models.

### 5.1. Entropy Generation Accumulation Analysis in Fatigue Process

**Figure 8:**
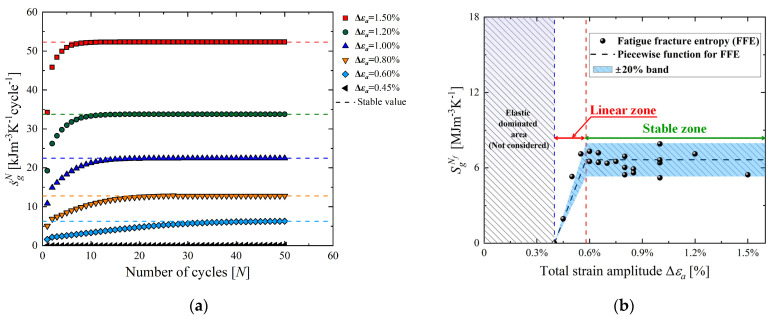
Entropy generation analysis. (**a**) Trend in cyclic entropy generation rate at different strain amplitudes for GH4169 at 650 °C. (**b**) Values of FFE versus different strain amplitudes at 650 °C.

This section first presents the entropy generation accumulation per cycle (cyclic entropy generation rate) in the LCF process for GH4169 at 650 °C in strain-controlled mode with load ratio *R_load_* = −1. Figure 8a presents the overall trend in cyclic entropy generation rate performed with different strain amplitudes ($\Delta \varepsilon_{a}=\Delta \varepsilon_{t}/2$). The trend indicates a gradual increase and eventually reaches a saturated state. The value for this saturated state can be defined as the stable cyclic entropy generation rate ${\dot{s}}_{g\_\mathrm{stable}}^{\;N}$, which is related to ($N_{i}/S_{g}^{\;N_{i}}$). Therefore, the entropy generation accumulation in the LCF process can be estimated by:

(60) $$S_{g}^{N_{i}}=\int_{0}^{N_{i}}{\dot{s}}_{g}^{N}\mathrm{dN}\approx N_{i}{\dot{s}}_{g\_\mathrm{stable}}^{N}$$

where *N_i_* is the number of cycles and $S_{g}^{N_{i}}$ is the FFE when $N_{i}=N_{f}$.

The experiment fatigue lives of GH4169 for modeling FFE ($S_{g}^{N_{f}}$) are listed in Table 2. These experimental fatigue lives were collected from a series of uniaxial strain-controlled fatigue tests at 650 °C with given loading ratio *R_load_* = −1 and frequency (1 Hz). Entropy generation accumulation versus different applied strain amplitudes is plotted in Figure 8a. It can be observed that there is a linear growth zone at low applied strain, and then the values of FFE floating within a ±20% band in a stable zone at a higher applied strain level.

It can be noted that FFE can only be treated as a material constant in the range of the stable zone. Thus, the opinion of Khonsari’s work for treating FFE as a material constant may not be appropriate for a wide range of applied load (stress or strain). The boundary between these two zones can be determined by the proportion of inelastic strain amplitude in stable stress–strain hysteresis loops, defined as β (=$\Delta \varepsilon_{\mathrm{in}}/\Delta \varepsilon_{a}$). According to the simulation results, the condition of the stable zone is satisfied when β ≥ 0.25. Also, the FFE for applied strain near the elastic limit is close to 0, which is too small compared to the FFE value of stable zone. When the strain is applied in elastic-dominant area, fatigue life increases and enters the high-cycle fatigue (HCF) category. In this category, the principal factors changed are internal friction, damping, or phase lag, as described in [36]. However, this area has not been investigated in this paper. Therefore, this paper proposes a piecewise model for FFE mainly aimed at LCF:

(61) $$S_{g}^{N_{f}}=\left\{\begin{array}{cc} A(\Delta \varepsilon_{a}-{\left[\Delta \varepsilon_{a}\right]}^{k_{0}}) & 0<\;\beta \leq 0.25\\ 6.641 & \;\beta >0.25 \end{array}\right.$$

where *A* is a material parameters and its value is equal to 3338 for GH4169 at 650 °C. ${\left[\Delta \varepsilon_{a}\right]}^{k_{0}}$ is the strain amplitude corresponding to initial yield stress (elastic limit) *k*_0_ and its value for GH4169 at 650 °C is about 0.003811. The average value of FFE in the stable zone is 6.641 MJm^−3^K^−1^. This method has the limitation that it is not applicable for HCF related to the elastic-dominant area, since the FFE for HCF is too small to be incorporated into the FFE model of LCF. Therefore, the FFE model for HCF requires further systematic thermodynamic research.

### 5.2. Influence of Load Ratio on Entropy Generation

**Figure 9:**
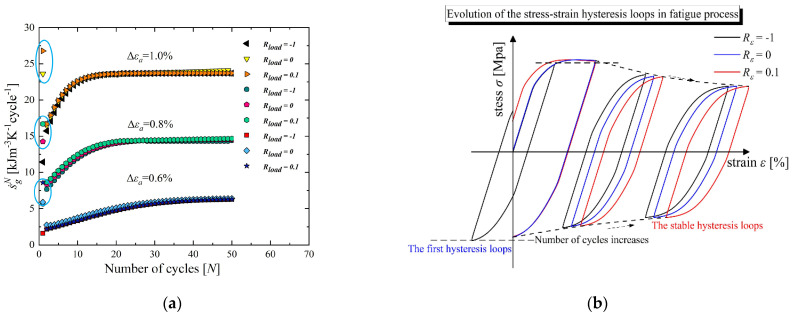
Entropy generation analysis considering different load ratios. (**a**) Evolution of cyclic entropy generation rate with various strain amplitudes. (**b**) Schematic diagram of the size evolution of the hysteresis loops from initiation to stabilization.

The influence of the load ratio *R_load_* is investigated in this section. The evolution of the cyclic entropy generation rate for the fatigue process performed with different *R_load_* values is first illustrated in Figure 8a. All the cyclic entropy generation rates eventually approach a constant state and the stable cyclic entropy generation rate ${\dot{s}}_{g\_\mathrm{stable}}^{N}$ performed with different load ratios can be assumed to be equal. However, the blue circle marked in Figure 9a indicates that the entropy generation accumulation in the initial cycle shows significant differences. Obviously, as the load ratio increases, the entropy generation accumulation in the initial cycle increases.

This phenomenon can be related to the intrinsic dissipation (Equation (42)) of the first cycle, and the degree of intrinsic dissipation is proportional to the size of the hysteresis loops, as shown in Figure 9b. It is observed that a larger area of the first hysteresis loops is associated with the increase in *R_load_*, causing larger macroscopic irreversible inelastic deformation and microscopic dislocation pileup with multiplication (mainly manifested as hardening/softening). Therefore, as the load ratio increases, the entropy generation accumulation in the first cycle increases, reflecting more severe irreversible damage.

**Figure 10:**
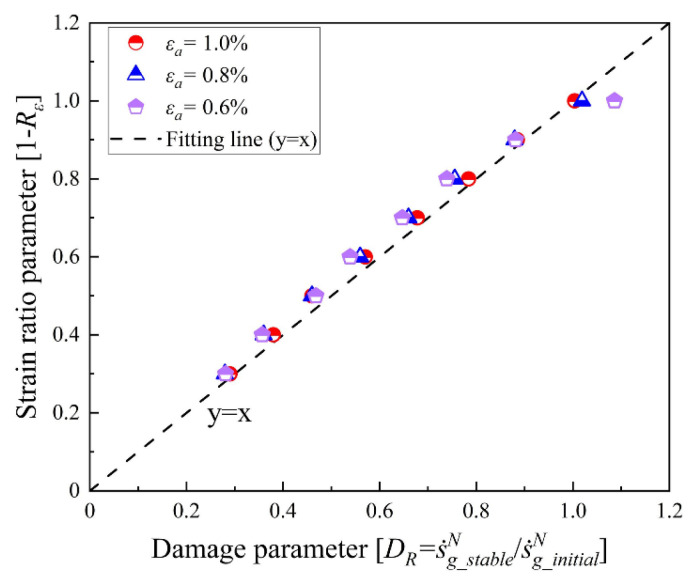
Relationship between damage parameter *D_R_* and load ratio parameter (1 − *R_load_*).

Therefore, the variation in load ratio significantly affects the entropy generation accumulation in the first cycle, which defines a variable called initial damage entropy generation ${\dot{s}}_{g\_\mathrm{initial}}^{N}$. This work utilizes the value of ${\dot{s}}_{g\_\mathrm{initial}}^{N}$ to characterize the initial fatigue damage for the further establishment of the life prediction model. Therefore, a novel damage parameter based on entropy generation called *D_R_* can be defined as:

(62) $$D_{R}=\frac{{\dot{s}}_{g\_\mathrm{stable}}^{N}}{{\dot{s}}_{g\_\mathrm{inital}}^{N}}$$

and the relationship between *D_R_* and the load ratio *R_load_* is clearly illustrated in Figure 10. According to the fitting line (*y = x*), Equation (62) can be estimated as:

(63) $$D_{R}=\frac{{\dot{s}}_{g\_\mathrm{stable}}^{N}}{{\dot{s}}_{g\_\mathrm{inital}}^{N}}≅1-R_{\mathrm{load}}$$

This is the expression considering the influence of the load ratio, and the role of this parameter is investigated in the next section.

### 5.3. Proposed Life Model Based on Thermodynamic Entropy Generation

As mentioned in Section 5.1, the fatigue failure life *N_f_*_:_ can be estimated by the linear function expressed by Equation (59). The stable cyclic entropy generation rate ${\dot{s}}_{g\_\mathrm{stable}}^{N}$ was introduced into this function, as follows:

(64) $$N_{f\;}=(\frac{N}{S_{g}^{\;N_{i}}})S_{g}^{\;N_{f}}\;≅\;(\frac{1}{{\dot{s}}_{g\_\mathrm{stable}}^{N}})S_{g}^{\;N_{f}}$$

The value of FFE $S_{g}^{\;N_{f}}$ can be estimated using Equation (61). Subsequently, considering the initial damage parameter (*D_R_*) based on entropy generation proposed in Section 5.2, a thermodynamic entropy-based model considering the influence of the load ratio is proposed:

(65) $$N_{f\;}=(\frac{1}{{\dot{s}}_{g\_\mathrm{stable}}^{N}})S_{g}^{\;N_{f}}(\frac{D_{R}}{2})$$

It can be noted that when *R_load_* = −1, Equation (63) can convert to Equation (64).

**Figure 11:**
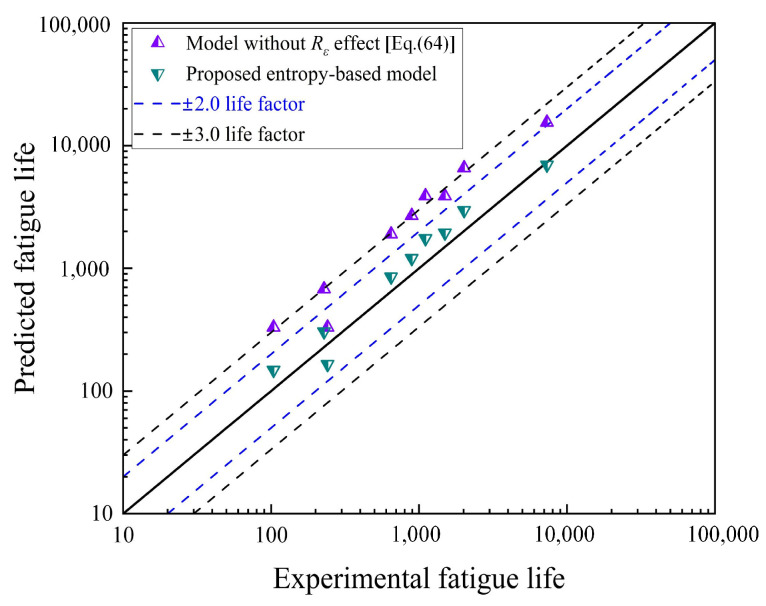
Experimental results versus predicted lives based on life model without load ratio effects and proposed thermodynamic entropy-based model (*R_load_* = 0 and 0.1) [51,52,53].

In order to validate Equation (65) with the influence of strain ratio, the predicted lives estimated by the proposed model were compared with the results from experiments performed with different asymmetric fatigue loading conditions (*R_load_* = 0 and 0.1), as shown in Figure 11. It was observed that all the predicted lives estimated by Equation (61) fell within the error band of 2 when the influence of the loading ratio was considered. Moreover, the proposed model improved the prediction accuracy by approximately 50% compared to Equation (64) without considering the loading ratio. Therefore, the proposed thermodynamic entropy-based model can better reflect the influence of loading ratio.

Finally, a series of fatigue lives are presented from LCF experiments as well as those predicted by employing the proposed entropy-based model and other classical life prediction models, such as the Manson–Coffin model, Ostergren energy model, Walker strain model, and SWT model. To demonstrate the contrast, all the parameters for these models were fitted from the same group of experimental fatigue lives listed in Table 2, as shown in Section 5.1. All the forms and parameters of these models are listed in Table 3. Obviously, the thermodynamic entropy-based model is the most convenient as it depends only on the single parameter *A*.

**Figure 12:**
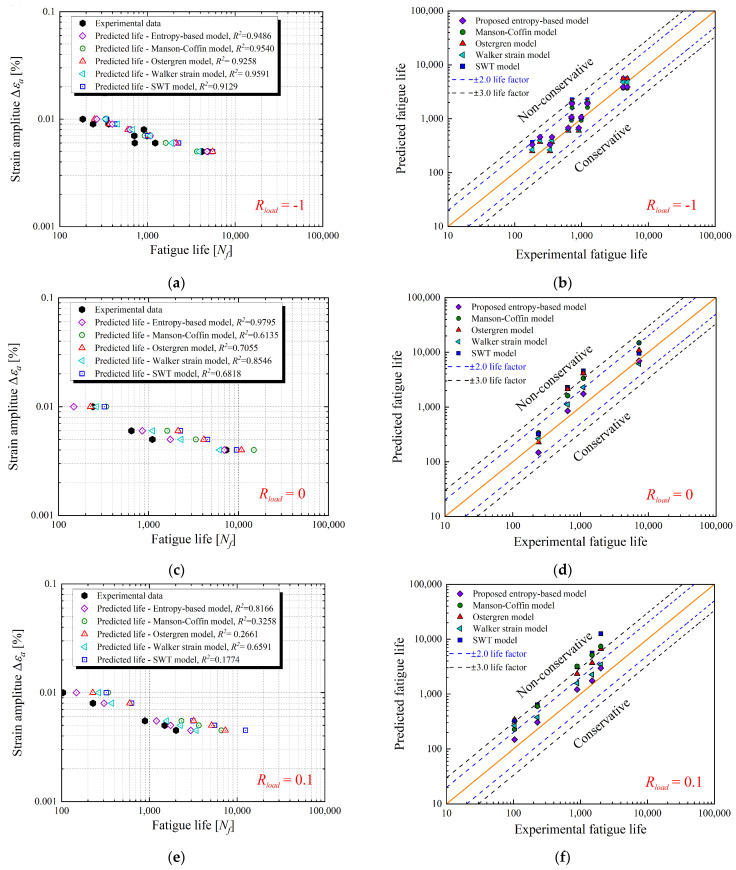
${∆\varepsilon }_{a}-N$ curves and scatter bands for the comparison between predicted fatigue lives and experimental data from fatigue tests. (**a**,**b**): *R_load_* = −1 [51,52,57]. (**c**,**d**): *R_load_* = 0 [53]. (**e**,**f**): *R_load_* = 0.1 [51,52].

Subsequently, groups of experimental fatigue life tested with different loading ratios (*R_load_* = −1, 0 and 0.1) were utilized to validate the accuracy of life prediction. Figure 12a–f graphically presents the comparison between the experimental results and the predicted life obtained by applying different life models. Therein, Figure 12a,c,e are the ${∆\varepsilon }_{a}\;-\;N$ curves and Figure 12b,d,f are the predicted life versus experimental life with scatter bands. Also, a common index *R*^2^ was introduced to clearly quantify the accuracy of different life models, which can be expressed as:

(66) $$R^{2}=1-\sum_{i=1}^{M}{\left(y_{\mathrm{ri}}-y_{\mathrm{pi}}\right)}^{2}/\sum_{i=1}^{M}{\left(y_{\mathrm{ri}}-y_{m}\right)}^{2}$$

where *y_pi_* and *y_ri_* are the predicted and actual values, respectively, *y_m_* is the average of *y_ri_*, and *M* represents the number of the fatigue lives. The *R*^2^ values corresponding to each life model are given in the legends of ${∆\varepsilon }_{a}-N$ curves for different loading ratios. As illustrated in Figure 12a,b, the proposed thermodynamic entropy-based model and other classical life prediction models can provide relatively close accuracy when *R_load_* = −1. Almost all the predicted lives can fall within the scatter band with a factor of 2 compared to the experimental results, and all the *R*^2^ values for each life model are within 0.9–0.96. However, for other classical life models, the fatigue lives are overestimated under asymmetric loading conditions (*R_load_* = 0 and 0.1), as shown in Figure 12c–f. Obviously, only the entropy-based model has the maximum value of *R*^2^, and the deviations in its life predictions can all fall into the scatter band with a factor of 2. Thus, the proposed entropy-based model can provide the best prediction of lives compared to the other life models. Therefore, it can be concluded that entropy generation combined with the unified viscoplastic constitutive model can provide an effective way to evaluate fatigue life.

## 6. Conclusions

The LCF fatigue life for GH4169 at 650 °C can be estimated based on entropy generation combined with the Chaboche unified viscoplastic constitutive theory. A damage parameter *D_R_* was proposed based on initial cyclic entropy generation ${\dot{s}}_{g\_\mathrm{inital}}^{N}$ to represent initial fatigue damage. Based on this parameter, the influence of load ratio was introduced to establish a novel entropy-based life prediction model. The conclusions drawn are as follows.

(1) The cyclic entropy generation rate is approximately a constant value when the cyclic stress–strain response is stable. The entropy generation accumulation during fatigue life, called fatigue fracture entropy FFE ($S_{g}^{\;N_{f}}$), can be calculated with the stable cyclic entropy generation rate and estimated by a piecewise FFE model related to the applied strain in the LCF category.
(2) Entropy generation under different loading ratios was investigated, and the initial cyclic entropy generation rate ${\dot{s}}_{g\_\mathrm{inital}}^{N}$ is the main difference. A damage parameter *D_R_* based on ${\dot{s}}_{g\_\mathrm{inital}}^{N}$ was defined to represent this difference and introduce the effect of load ratio in LCF.
(3) A thermodynamic entropy-based model with the damage parameter *D_R_* was proposed to estimate fatigue life. The predicted results from the proposed model show good concordance with the experimental results. Compared with the classical models, such as Manson–Coffin, Ostergren, Walker strain, and SWT, the results indicated that the proposed model can provide better prediction accuracy with higher *R*^2^ and smaller dispersion.

## Figures and Tables

**Table 1:** Viscoplastic constitutive model parameters of GH4169 at 650 °C [43].

Viscoplastic	*Z* = 893 MPa∙s^1/*n*′^	*n*′ = 3.9	*k*_0_ = 678 MPa
Elastic	*E* = 171.6 GPa		
Isotropic hardening	*Q* = −380 MPa	*b* = 13.2	
Kinematic hardening	*C*_1_ = 495	*a*_1_ = 179 MPa	
	*C*_2_ = 350	*a*_2_ = 187 MPa	

**Table 2:** Fatigue lives collected from GH4169 isothermal fatigue tests at 650 °C to determine FFE ($S_{g}^{N_{f}}$) [56].

$\mathrm{Strain}\;\mathrm{Amplitude}\;\Delta \varepsilon_{a}$(%)	Fatigue Failure Life *N_f_* (Cycles)
1.50%	104
1.20%	213
1.00%	295
	279
	231
	354
0.85%	395
	378
0.80%	541
	471
	426
0.75%	589
0.70%	592
0.65%	837
	934
0.60%	1036
	1194
0.575%	2027
0.50%	3976
0.45%	15,497
0.40%	130,585

**Table 3:** Fatigue life prediction models for GH4169 alloy at 650 °C.

Life Prediction Models	Parameters Fitting from Test Data in Table 2			
Manson–Coffin model	$\varepsilon_{f}^{\prime }$	*c*	$\sigma_{f}^{\prime }$	*b*
$\frac{\Delta \varepsilon_{t}}{2}=\varepsilon_{f}^{\prime }{\left(2N_{f}\right)}^{c}+\frac{\sigma_{f}^{\prime }}{E}{\left(2N_{f}\right)}^{b}$	0.5771	−0.727	1423	−0.079
Ostergren energy model	*M*	*C*		
$\left(\sigma_{\max}\Delta \varepsilon_{\mathrm{in}}\right){N_{f}}^{M}=C$	0.538	170.65		
Walker strain model	*m*	*u*	*v*	
$\frac{\sigma_{\max}}{E}{\left(\frac{\Delta \varepsilon_{t}}{\sigma_{\max}/E}\right)}^{m}=u{\left(N_{f}\right)}^{-v}$	0.8020	0.0477	0.2130	
SWT model	$\varepsilon_{f}^{\prime }$	*c*	$\sigma_{f}^{\prime }$	*b*
$\frac{\sigma_{\max}\Delta \varepsilon_{t}}{2}=\sigma_{f}^{\prime }\varepsilon_{f}^{\prime }{\left(2N_{f}\right)}^{b+c}+\frac{{\left(\sigma_{f}^{\prime }\right)}^{2}}{E}{\left(2N_{f}\right)}^{2b}$	0.5771	−0.727	1423	−0.079
Thermodynamic entropy-based model	*A*			
$N_{f}=(\frac{1}{{\dot{s}}_{g\_\mathrm{stable}}^{N}})S_{g}^{N_{f}}(\frac{1-R_{\mathrm{load}}}{2})$ $S_{g}^{N_{f}}=\left\{\begin{array}{cc} A(\Delta \varepsilon_{a}-{\left[\Delta \varepsilon_{a}\right]}^{k_{0}}) & 0<\;\beta \leq 0.25\\ 6.641 & \;\beta >0.25 \end{array}\right.$	3338			

## Data Availability

The data that support the findings of this study are available from the corresponding author upon reasonable request.
